# Putting Evolution to Work: Host Range Alteration for Phage Therapy

**DOI:** 10.3390/v18080871

**Published:** 2026-08-10

**Authors:** Rachel Stanchek, Paul Hyman

**Affiliations:** Department of Biology & Toxicology, Ashland University, Ashland, OH 44805, USA; rstanche@ashland.edu

**Keywords:** phage therapy, bacteriophage, host range, receptor binding protein, evolution, mutation, genetic engineering, antibiotic-resistant bacteria

## Abstract

Host range describes the hosts that a bacteriophage can and cannot infect. Throughout the history of phage use in various applications, phages with varying host ranges have been needed and techniques to alter a phage’s host range have been developed. These include techniques that rely on spontaneous mutations and others that use genetic engineering to make designed changes. Phages with altered host ranges have been used in personalized phage therapy cases and in cocktails in clinical trials. In this review, we provide descriptions of methods for altering a phage’s host range with a focus on applications to phage therapy.

## 1. Introduction

A bacteriophage’s (phage’s) host range is an intrinsic property that describes which hosts it can successfully infect. For this review, we will mainly use the term host range to refer to a bacteriophage’s productive host range, which means the hosts that it can infect and produce progeny phages from [1]. In this sense, the host range for lytic infections depends on (a) successful binding of the phage to the host cell; (b) genome entry and infection establishment including surviving host defenses such as restriction enzymes and CRISPR-Cas endonucleases; (c) bypassing later acting host defenses such as toxin-antitoxin and abortive infection mechanisms while producing and assembling progeny viruses; and (d) release of progeny virions. Failure to complete any of these steps in a particular host strain or species would mean that the strain or species is outside the phage’s host range.

For phage therapy applications, host range determines if a phage will be useful in treating a particular infection [2,3]. Only strains of targeted bacteria within a phage’s host range will be infected and killed. Strains outside the phage’s host range are immune. For personalized phage therapy, it is necessary to test the phage(s) on the patient’s infecting strain to confirm that it is within the phage’s host range [4]. For phages being developed for use in fixed cocktails (mixtures of multiple phages) meant to treat many infected individuals without strain testing, the most useful ones are those with broader host ranges, preferably overlapping [5,6]. Having more than one phage in a cocktail, ideally ones that use different receptors and different host defense bypass mechanisms, should help prevent or slow down the development of phage-resistant bacteria [2,7].

While it is relatively easy to isolate phages for some hosts, other hosts are more challenging [8,9]. In some situations, rather than isolating a novel phage with the appropriate host range, it may be simpler to alter the host range of a previously isolated phage so that it can infect a host outside its natural range. Other reasons for modifying a phage’s host range include

- Saving time and expense by using well characterized phages rather than newly isolated phages;
- Making broader host range phages for cocktails to increase the overall strain/species coverage of the cocktail;
- Keeping up with host evolution and overcoming bacterial resistance to phage infection.

A number of methods have been developed to alter the host range of phages. These are summarized in Table 1. Broadly, these can be divided into ones that rely on spontaneous mutations and ones that rely on genetic engineering of phages. In the former category are procedures such as phage adaptation and the Appelmans protocol. In the latter category, phages may be modified by site-directed mutagenesis, swapping of tail fiber domains and the insertion of genes that protect the phages from host defense systems. In this review, we will provide an overview of these methods, including their current use in phage therapy.

## 2. Phage Adaptation

Outside of the laboratory, bacteriophages, like other parasites, must evolve to be able to continue infecting hosts even as the hosts evolve protection mechanisms [10,11,12], which can include host range shifts. This is not a new observation. Felix d’Herelle describes experiments showing that some strains of phages capable of killing one strain of *Bacillus typhosus* (now *Salmonella enterica* serovar Typhi) would, after multiple passages on a previously resistant strain, be able to efficiently kill that and additional strains of *B. typhosus* [13]. This process of host range expansion, which relies on random mutation, came to be called phage adaptation. As it came to be practiced, phage adaptation is simply the isolation of rare host range mutants from a population of phages by exposing bacteria that are not within a phage’s host range to a large number of phages (e.g., 10^7^ per infection) and looking for growth. It has been used for generating phages with altered host ranges for a variety of purposes over time.

One early use of phage adaptation was to isolate new strains of a bacteriophage to use in the phage typing of bacteria [14]. Phage typing is the use of a standardized panel of bacteriophages to sort strains of a bacterial species into groups that have the same pattern of susceptibility and resistance to the bacteriophage panel [15]. First developed by Craigie and colleagues [16,17], phage typing remained useful for tracking strains involved in epidemic outbreaks for many years, and collections of phages for particular pathogens were increased to detect and distinguish new strains of pathogens (e.g., [18]). Most recently, it has been largely supplanted by molecular biology techniques (e.g., pulse-field gel electrophoresis (PFGE)) and whole genome sequencing [19].

Smith [20] adapted phages for *Staphylococcus* typing by spotting phages onto a spread lawn of bacteria on a plate and looking for plaques. Wilson and colleagues [21] adapted phages by inoculating cultures of *Salmonella typhimurium* with a phage that did not normally infect those strains, incubating overnight, heat-killing bacteria, and centrifuging to remove bacterial debris. The resulting supernatant was screened for phage plaques on the same bacterial strain. Phage adaptation to create typing phage has been done with phage-killing *Clostridium perfringens* [22]; *Mycobacterium fortuitim* [23]; and *Propionibacterium acnes* [24] among many other species.

More recently phage adaptation has been used to study host range expansion in the context of creating or improving phages for phage therapy. The term phage adaptation has stopped being used in favor of spontaneous mutation (or just mutation), but the procedure of isolating phages with improved plaque formation on previously resistant hosts remains the same. For example, He and colleagues [25] isolated a mutant phage infecting *Acinetobacter baumannii* and found that the host range altering mutation was in a tail tube protein rather than the more common receptor binding protein. Similarly, Boon and colleagues [26] isolated mutant *Pseudomonas* LUZ7 phages that had an expanded host range growing on the previously partly resistant PAO1 strain as indicated by a plaque morphology change from turbid to clear. They sequenced several of these mutant phages and found they all carried an identical mutation in a tail fiber gene. Phages with this mutation were shown to adsorb more efficiently to the host bacteria. Sergueev and colleagues [27] also used phage adaptation to generate phages able to infect a wider range of clinical isolates of methicillin-resistant *Staphylococcus aureus* (MRSA). Dedrick and colleagues [28] used a cocktail of three phages to treat a patient with disseminated-drug-resistant *Mycobacterium abscessus*. One of the phages was described as a host range mutant of a phage that only poorly infected *M. abscessus*.

## 3. Appelmans Protocol

The Appelmans protocol [29] is derived from a liquid dilution method which was found to expand phage host range after multiple passages [30]. Unlike phage adaptation, the Appelmans protocol starts with a cocktail of bacteriophages and produces a cocktail with an expanded host range. Often, this includes infecting and killing bacteria that were previously resistant to the cocktail [31]. In this, the Appelmans protocol expands the cocktail’s host range to include a specific set of bacterial strains, although the new host range may also include other strains as well. Individual phage strains can be isolated from the cocktail and these can have altered host ranges as well. This protocol has been used at the Eliava Institute of Bacteriophage, Microbiology and Virology (Tbilisi, Georgia) to maintain phage cocktail efficacy for many decades but has only recently been “rediscovered” by other phage therapy researchers.

Briefly, this method takes a cocktail of phages and uses it to individually infect strains of a host that range in sensitivity to the cocktail from fully sensitive to partially resistant to fully resistant. This is done using a serial dilution of the cocktail mixed with a culture of each host. After an overnight incubation, phages from the highest dilution that clears the bacteria of each strain plus one more dilution are collected, pooled and reapplied to all of the hosts for another round of growth. This is repeated until a desired percentage of the hosts are fully sensitive to the cocktail. According to Burrowes and colleagues [31], for example, “In the original Appelmans protocol the endpoint is reached when the output cocktail lyses >80% of the host strains at a minimum dilution of 10^−7^”, but additional rounds may continue to expand the host range of the cocktail further.

In contrast to phage adaptation, the Appelmans protocol is not always the result of mutations of the receptor binding proteins in the parent phages. Burrowes and colleagues [31] used three *P. aeruginosa* phages and 10 *P. aeruginosa* strains, eight of which were phage-resistant, to develop a new cocktail mixture able to kill all ten hosts and to study the mechanisms generating the novel phage cocktail. In parallel, they also exposed individual phages to the bacteria and failed to obtain any that had a similar increased host range. This suggests that mutation alone was not the source of the expanded host range phages. When they sequenced individual phage strains isolated from the broad host range cocktail, they found that the phages had undergone extensive recombination but almost no point mutations. The production of mosaic phages seems to better support host range expansion than mutation. This is not a universal mechanism, however. Lossouarn and colleagues [32] created a cocktail of four phages infecting *Enterococcus faecium* and used the Appelmans protocol with 15 passages against eight *E. faecium* strains. They isolated phages from the final passage and found that many had an altered or expanded host range from the four input phages. Sequencing some of these phages showed that there was some recombination but that there were also mutations that clustered in the tail fiber genes. They concluded that the mutations, rather than the recombination events, drove the expanded host range.

Some workers have found that the Appelmans protocol can trigger induction of cryptic prophages that can contribute to the cocktail’s expanded host range. Jakob and colleagues [33] used three phages infecting *Klebsiella pneumoniae* for 30 cycles of passaging. They did obtain a cocktail that had an expanded host range and isolated individual phages from that cocktail. They did not find any mosaic phages, but they did find a novel phage that turned out to be an induced cryptic prophage. Peters and colleagues [34] also found that a *P. aeruginosa* phage cocktail that had its host range expanded contained a novel bacteriophage from an induced prophage.

While not widely used, the Appelmans protocol has been used with several different phage–host combinations to improve the performance of phage cocktails.

- Mapes and colleagues [35] used the protocol to expand the host range of a cocktail of four *Pseudomonas aeruginosa* phages using 16 different *P. aeruginosa* strains. After 30 cycles, they isolated individual phage strains and found that they all had expanded host ranges on a panel of 25 *P. aeruginosa* strains. They made several combinations of these new phages and also showed that they were effective at preventing *Pseudomonas* biofilm formation.
- Kolenda and colleagues [36] used two separate cocktails to improve infection host range on eight *Staphylococcus aureus* strains. Only one of the cocktails improved its host range after 30 cycles of the Appelmans protocol. They sequenced several phage strains from the improved cocktail and found that they were composed of mosaics of two of the three ancestor phages in the original cocktail. Interestingly, they also found a cluster of mutations in several phage genes which suggested that the increased host range may have been due to an improvement in the phages’ ability to overcome bacterial anti-phage mechanisms rather than improved receptor binding.
- Vu and colleagues [37] made a cocktail of three phages infecting *Acinetobacter baumanii* and used the Appelmans protocol with ten carbapenem-resistant *A. baumanii* strains to improve the host range of the cocktail. They obtained an improved cocktail and isolated individual phages that were genome-sequenced. Surprisingly, none of the new phages were descended from the original input phages. Instead, they were recombinants of induced prophages present in the development bacteria.

The Appelmans protocol has been adapted for other uses as well. Kok and colleagues [38] modified the Appelmans protocol to increase the titer and host range of newly isolated phages infecting *Paenibacillus larvae* bacteria, a honeybee pathogen. They isolated 26 novel phages but none grew well, only achieving titers of 10^5^ to 10^7^ pfu/mL. After trying several methods that were not successful, they adapted the Appelmans protocol by only mixing a single phage-bacteria combination in each row of 96-well plates. They completed 30 cycles and found that phages had improved titers, plaque sizes, and host ranges. Since only a single phage was used, the improvements were most likely due to mutations but it was impossible to confirm this as the original phage strains’ titers were too low to provide enough DNA for sequencing. Still, it might be more accurate to describe this as a phage adaptation protocol done in 96-well plates.

Another related method, described by Salazar and colleagues [39] as utilizing a principle that “is similar to the original Appelman’s protocol for facilitating growth of phage on hosts refractory to predation,” uses continuous culture in a modified chemostat as opposed to 96-well plate batch cultures. In a recently issued patent which provides more details about this approach, they designate this modified chemostat a “Tetrastat” [40]. To test the method, a single phage, φHP3, was inoculated into a mix of a phage-sensitive and phage-resistant bacterial strains of pathogenic *E. coli*. Phages were isolated at various times after inoculation. A phage able to infect the resistant host was isolated from a 5 h post inoculation sample. Subsequent genome sequencing showed that the new phage was derived from the original φHP3 phage strain but had two point mutations. One mutation was in the spike protein gene and the other in was in the long tail fiber gene. As with the variant protocol of Kok and colleagues described in the previous paragraph, the use of a single input phage better aligns this method with phage adaptation methods than the Appelmans protocol.

## 4. Genetic Engineering of Phages

In addition to taking advantage of mutation-caused, random changes in host range, some phage researchers have used genetic engineering techniques to introduce specific changes into bacteriophages to alter their host range. These changes can be divided into three types—(a) changes in the receptor binding protein to affect host range by allowing phages to bind to different hosts; (b) addition of genes that provide novel killing mechanisms to phages; and (c) addition of other genes to allow phages to resist bacterial anti-phage mechanisms such as restriction enzymes or CRISPR-Cas systems.

### 4.1. Genetic Engineering Systems

The ease of genetically modifying phages depends on the phage-host system. There is a robust set of tools for working with *E. coli* phages but less so with other hosts. Researchers have developed a variety of techniques and tools for these systems ranging from classical gene cloning methods to cell-free rebooting systems. Table 2 lists some commonly used methods.

Additional reviews of these methods and others can be found in [2,55,56,57].

### 4.2. Receptor Binding Protein Changes

Early studies on the receptor binding proteins (RBPs) of bacteriophages showed that parts of these proteins could be swapped between bacteriophages by genetic recombination and that the resulting hybrid phage would have changed host range [58,59]. Because RBPs are usually at the C-terminal tip of tail fibers of tailed phages, the terms RBP and tail fiber are sometimes used interchangeably. The advent of methods of genetic engineering allowed for more specific targeting of bacteriophage portions of tail fibers, specifically focusing on specific receptor binding structures within the proteins. These methods have successfully expanded bacteriophage host range and could be potentially useful in the context of phage therapy. Broadly we can group the kinds of modifications made to (a) studies that replaced the receptor binding region with the equivalent sequence from another phage; (b) studies that inserted novel sequences into the phage receptor binding region, and (c) studies that inserted a library of varying sequences into the receptor binding region. Generally, the resulting hybrid (also called chimeric) phages were then screened for host range changes, although studies vary in the number of strains tested, making it unclear if the host range was expanded or just changed.

#### 4.2.1. Receptor Binding Region Exchange

The goal of exchanging RBPs is to replace the host range of a narrow-host-range phage with the host range of another phage that infects more hosts but may be unsuitable for phage therapy for other reasons such as being temperate or carrying a toxin gene. The RBP of the unsuitable phage can be cloned into a plasmid, and then the plasmid-carrying cell is infected with the narrow-host-range phage. Recombination will exchange the RBPs at some, often low, frequency. The resulting phages are then screened for ones with the new host range. The recombination frequency will depend on the amount of homology between the two phage RBPs but with a selective host; even rare recombinants can be identified. Multiple studies have successfully demonstrated that gene exchange can expand bacteriophage host range.

- Tetart and colleagues [60], while studying the RBP of *E. coli* phage T4, cloned the RBPs of *Yersinia* phages SV76.3 and Mi. Both of these phages infect *Yersinia pseudotuberculosis* which is not normally infected by T4 phage. While the original T4 phage only infects *E. coli* (and closely related species of Shigella), the recombinant phages were also able to infect *Yersinia*, showing a clear expansion of host range in the recombinant phage as compared to its parent phage.
- Mahichi and colleagues [61] generated 10 chimeric phages by homologous recombination of T2 phages and a plasmid which contained cloned copies of the long tail fiber genes of phage IP008. These recombinant phages had the broader host range (nine of 10 strains) of IP008 compared to the narrower host range (four of 10 strains) of T2 without any loss of lytic activity. They also found that these recombinant phages recognized the same receptor on the host cell surface as phage IP008, and not phage T2, based on patterns of infection of phage-resistant bacteria.
- Le and colleagues [42] studied expanding host range in phages targeting *P. aeruginosa.* Sequencing two phages, they found that host specificity is determined by the C-terminal regions of the tail fiber. They moved the tail fiber gene from an expanded host range mutant of phage JG004 into phage PaP1. The resulting recombinant phage had an expanded host range compared to the parental phage.
- Ando and colleagues [53] demonstrated the utility of the reassembly of phage genomes using yeast and native yeast gap repair for assembly of overlapping genome fragments by creating a series of T7-like phages with swapped and hybrid tail fibers. This let them modify the phage genome during synthesis of the genome fragments, bypassing the need to clone the RBPs or rely on recombination to introduce RBP mutations into the phage genome. Using phages that infected *E. coli* (T7 and T3), *Klebsiella* (K1E, K1F, K1-5, K11), *Yersinia pseudotuberculosis* (R), *Pseudomonas aeruginosa* (LUZ19), and *Pseudomonas putida* (gh-1), they used gene swapping of the C-terminal domains of the tail fiber to create a series of hybrid phages that had altered host ranges. Ando and colleagues suggest that this system can be adapted to other phage systems as a general strategy to expand a phage’s host range.
- Chen and colleagues [41] sequenced two different phages, WG01 and QL01 to identify their host-determinant gene regions in their RBPs. Different portions of this region in the QL01 gene were inserted into the WG01 phage genome using homologous recombination with cloned segments of the RBP. They isolated four different WG01 recombinant derivatives that had a wider host range than the original WG01 phage. In a follow-up study, Li and colleagues [62], showed that the WGqlae phage, derived from QL01 and WG01, was able to lyse additional hosts compared to its parent phages WG01 and QL01. WGqlae was also effective in inhibiting biofilm formation and clearing extant biofilm of *E. coli.*
- Zhang and colleagues [63] isolated two T5-like phages infecting *Salmonella*—a narrow-host-range phage, STyj5-1, and a wide-host-range phage, BD13. Parts of the long tail fiber (pb1) of STyj5-1 were replaced by those of BD13 using homologous recombination to create several hybrid phages. Some of these hybrid phages displayed an expansion of the STyj5-1 phage host range. These recombinant phages were able to lyse 10 additional strains compared to the STyj5-1 phage and had the same host range as the BD13 phage including growth on *E. coli* strains.
- Zheng and colleagues [64] replaced the long-tail fibers genes (PB3 and PB4) of the *Salmonella* T5-like phage PH204, which had a narrow host range, with the tail fiber genes of a wider host range bacteriophage, SP76. They accomplished this by cloning the SP 76 genes using PCR and using recombination to move the cloned genes into PH204. This produced two novel T5-like phages with an extended host range. The novel T5-like phages could lyse 54 of 97 *Salmonella* strains as compared to the parent PH204 phage which could only lyse 30 strains. These new phages retained other biological characteristics of the parent phage.

#### 4.2.2. Receptor Binding Region Redesign by Inserting Novel Sequences

Improvements in genome sequencing and protein structure analysis have allowed some researchers to make specific changes to RBPs before transferring the gene into a phage genome.

Dunne and colleagues [51] analyzed the structure of the receptor binding protein of *Listeria monocytogenes* phage PSA and used that information to create phages with altered host range. First, they cloned the RBP gene and used error-prone PCR to create a library of mutant genes that were inserted into phage and screened for altered host range. Second, they used these mutant RBP genes to create polyvalent phages that contained both the wild-type and mutated RBP genes. Third, they determined the crystal structure of the identified mutant RBPs and used that information to design and create novel chimeric RBPs. These chimeric RBPs included different combinations of what they called the “head”, “neck” and “tail” regions of the RBP. The chimeric RBP genes were used to replace the RBP gene in five different phages that combined prophage capsids with chimeric RBPs. The resulting phages displayed the host range corresponding to the RBP used. The authors propose that these methods could be applied to other phage-host systems to produce phages that are more useful for phage therapy than native phages.

#### 4.2.3. Receptor Binding Region Libraries

The previously cited studies all created populations of phages that had the same RBP. It is possible, however, to insert a library of RBP changes into phages and grow populations of phages that contain all of the altered RBPs. M13 phages with libraries of peptides inserted into the receptor binding protein have long been used for phage display, a method to select peptides with specific binding capacities [65]. For phage display, the peptide insertion site does not interfere with the host receptor binding. In contrast, a few groups have inserted or mutated RBP sequences to create libraries of phages that have altered host ranges by changing the receptor binding sequences. This includes Dunne and colleagues [51] who created a library of phages, as well as redesigned tail fiber binding domains based on the structure of native domains (described above).

- Yehl and colleagues [66] studied the structure of the T3 RBP as well, sequencing multiple mutant phages that could infect *E. coli* T3-resistant mutants. With this information, they identified several amino acid loops at the tip of the T3 RBP that were the likely sites of receptor-RBP contact. They used two PCR methods with degenerate primers to introduce mutations in all the identified loop sites. The mutated RBP segments were reintroduced into the phage by phage–plasmid recombination to create a library of phages with different binding specificities. This procedure produced a high diversity cocktail of phages differing by one or a few amino acids in the RBP. This cocktail was better able to suppress the appearance of phage-resistant bacteria than single phage. They could also isolate individual phages from the cocktail that had improved host range on a panel of T3-resistant *E. coli.* Finally, they tested one of isolated phages, PB10, at killing bacteria in vivo using a mouse skin model and found that PB10 outperformed the wild-type phage.
- Liang and colleagues [54], using a chemically synthesized *E. coli* phage T7 genome and a cell-free transcription-translation system to produce phages from the synthetic genome, inserted a library of phages with inserts in the RBP gene and other genes. They also created a T7 genome with a mutagenized library of genes based on the RBP of phage T3. These libraries contained phages with altered host range on *E. coli* and also ones that could infect species of *Salmonella* and *Yersinia*. They noted that the use of synthesized genomes and cell-free transcription-translation systems could have wide applicability to other phage–host combinations.
- Song and colleagues [49] developed a Cas9-assisted recombineering method to expand host range with precise substitutions in the tail fiber of *E. coli* phage PHB20, a T7-like phage. They inserted a small library of 30 tail fiber segments into the phage and obtained 30 hybrid phages. The tail fiber segments were obtained by PCR amplification of phages in environmental water samples. While they did not broadly test host range, the recombinant phages that were produced were able to infect both *E. coli* BL21 and an E22 strain while the parent phage could not infect the E22 strain, suggesting that the recombinant phages had expanded host ranges in comparison to the parent phage.
- Huss and colleagues [67] created a library of about 5000 different T7 phages with single amino acid substitutions in the tip of the phage tail fiber protein. They cultured this library on *E. coli* BL21 for 23 days in the lab and in cultures in microgravity at the International Space Station. Both cultures were enriched for particular variants but not the same variants. Although they did not examine host range, a cocktail of the 13 most frequent variants from the microgravity enrichment showed a higher efficiency of plating and increased plaque size growing on BL21 in the laboratory while a similar cocktail of the lab selected variants did not. The researchers suggest that both changes in bacterial physiology and phage-bacterial collision dynamics due to the microgravity environment may have played a role in the differences they observed, but also suggested that more experimentation will be needed to identify more mechanistic details.

### 4.3. Engineered Phages Containing New Genes

Phages that have been modified to be more effective at killing bacteria due to the addition of new genes are sometimes described as “armed” phage [68,69]. This arming can expand host range or provide phages with novel bacterial killing mechanisms such as capsid-bound photosensitizers [70] or nanoparticles [71,72]. For this section, we will describe genetic engineering that inserts novel genes that are expressed during phage infection. Other review articles [57,68,69] contain descriptions of phages that have attached bacterial-killing moieties that act without any phage gene expression.

#### 4.3.1. Depolymerases

One set of novel genes used are depolymerases, from phages or other species, that break down bacterial extracellular matrix molecules, commonly polysaccharides, that are found in bacterial capsules and biofilms. These molecules protect bacteria from phage infection by masking phage receptors. The addition of depolymerase genes to phages allows the phages to infect bacteria that would be otherwise outside of the phages’ host range. Lu and Collins [73], for example, created a hybrid version of phage λ that had a gene for dispersin B (*DspB*) from *Actinobacillus actinomycetemcomitans.* This enzyme can break down biofilms of *E. coli* and *Staphylococcus.* They also added the T3 phage gene 1.2 whose product also promotes growth on biofilm-forming *E. coli* that contain an F-plasmid. F-plasmids contain genes that support biofilm formation. The hybrid phage was able to degrade over 99% of biofilms formed by *E. coli*.

Hupfeld and colleagues [74] created a CRISPR-Cas-based cloning system in *Listeria* and used it to add a lysostaphin gene (from *Staphylococcus simulans*) to the genome of the *Listeria* phage A511. This lysostaphin specifically acts on *Staphylococcus*, not *Listeria.* When they infected a mixed culture of *Listeria* and *Staphylococcus* both bacteria were killed—the *Listeria* by the phage and the *Staphylococcus* by the lysostaphin released by infected cell lysis. This shows that a single genetically modified phage could be used to specifically target two types of bacteria, perhaps for use in targeted microbiome modification. Similarly, Kilcher and colleagues [52] used a novel phage genome reassembly method to replace the endolysin of *Listeria monocytogenes* phage PSA with a more broadly functioning endolysin from phage A511. The resulting hybrid phage was able to infect PSA-resistant *L. monocytogenes*.

#### 4.3.2. CRISPR-Cas Genes

Another set of genes that have been added to phages to enhance their killing of bacteria are CRISPR-Cas genes. The bacterial CRISPR-Cas system consists of two elements: CRISPR arrays of DNA sequences that function as the targeting elements via the guide RNAs that they encode, and Cas genes that effect the DNA cleavage and resulting target-organism-killing. The *Cas9* and *Cas3* genes are commonly used as both are able to cleave double-stranded DNA [75]. Phages with CRISPR-Cas added do not have altered RBPs but still can be considered to have altered host range because they are able to kill cells (via genome cleavage) that are outside the phages’ productive host range due to host restriction or metabolic incompatibility, for example. Another benefit of this approach is that temperate phage can be used because the CRISPR-containing temperate phage kills the host cell soon after infection before the phage can integrate its genome and establish the lysogenic cycle [76].

Two early examples of this approach used phagemid vectors to add Cas genes and CRISPR targeting arrays to filamentous phages. Bikard and colleagues [44] used the *Streptococcus pyogenes* Cas9 gene and a targeting CRISPR array containing sequences specific to antibiotic-resistant *Staphylococcus aureus.* These were inserted into phagemid pC194 which produces the *Staphylococcus* phage φNM1. The modified phages selectively killed antibiotic-resistant *S. aureus* in vitro and in vivo using a mouse skin colonization model. Similarly, Citorik and colleagues [45] developed an M13-based CRISPR-Cas phage that contained a targeting array directed at antibiotic-resistant *E. coli*. These phages were tested in vivo using a *Galleria mellonella* larva infection model. They found that the armed phages significantly improved larva survival.

More recently, Gencay and colleagues [77] created a cocktail of four coliphages from a pool of newly isolated phages by: (a) swapping some of the RBP domains using genetic engineering techniques; (b) using phage adaptation to select for improved infection efficiencies of phages; and (c) adding Cas3a genes with an *E. coli* CRISPR targeting array to the phages. The cocktail, designated SNIPR001, showed good activity, with positive spot tests on over 95% of a panel of 429 clinical *E. coli* isolates. At higher doses, the cocktail was effective at suppressing *E. coli* growth using a single-strain colonized mouse model. The cocktail was also well-tolerated in minipigs and phages could be recovered in gastrointestinal track samples. Based in part on this preliminary data, the cocktail was used in a phase 1, placebo-controlled, double-blind safety trial [78]. Thirty-six healthy individuals were recruited into three cohorts and received a seven-day oral administration of the phage cocktail or a placebo control. The phage cocktail was well-tolerated by the recipients with fewer adverse effects in the experimental cohort compared to the placebo group. The normal *E. coli* microbiome was also relatively unaffected by the treatment. The eventual goal is to use the SNIPR001 cocktail to treat patients with *E. coli* infections and patients who develop bacteremia during treatment for hematological cancer. This cocktail is being developed by SNIPR Biome (Copenhagen, Denmark) who also has other cocktails for treating other infections that are still in the development phase (https://www.sniprbiome.com/pipeline (accessed on 15 July 2026)).

LBP-EC01 is another cocktail that has recently undergone clinical trial testing [79]. It is a cocktail of six phages, three of which have had Cas3 genes added along with CRISPR arrays targeting conserved *E. coli* genome sequences. The cocktail was developed to treat urinary tract infections and infected 96% of a panel of 356 uropathogenic *E. coli* strains. A phase 2/3 clinical trial with the cocktail is currently recruiting patients (https://www.eliminateuti.com/ (accessed on 15 July 2026)). Earlier phase 1 results showed that the cocktail is well-tolerated in a group of 39 patients with uncomplicated UTIs [79]. The phase 1 results were also used to establish dosing and other parameters for the current trial. This cocktail is being developed by Locus Biosciences (Morrisville, NC, USA) who is also developing cocktails for treating *Pseudomonas aeruginosa*, *Klebsiella pneumoniae*, and *Staphylococcus aureus* that are still in the pre-clinical phase (https://www.locus-bio.com/programs/ (accessed on 15 July 2026)).

Additional examples of Cas-armed phages developed for laboratory or clinical trial testing can be found in [56,80].

#### 4.3.3. Antimicrobial Peptides

Antimicrobial peptides (AMPs) are relatively short peptides made by animals and plants that have bactericidal activity. They are generally broad-spectrum and function by attacking cell membranes or cell walls [4,81]. Their direct use as antimicrobial agents is limited by their susceptibility to proteases, potential effects on mammalian cells (poor selective toxicity), and high synthesis costs in bulk. Several groups have added AMP genes to phages with the goal of making the phages and the AMPs more effective at killing bacteria. Because the modified phages must infect a host cell to express the AMP gene, these modifications have little effect on host range, although they can potentially allow phage to kill bacteria that would be outside the phage’s host range due to restriction or metabolic incompatibility. Some AMPs could potentially be used with temperate phages to kill host cells before lysogenic bacteria could be established. It is notable that host range change was not determined in studies that added AMP genes to phage.

- Krom and colleagues [82] tested multiple AMPs inserted in pairs to an M13 phagemid genome. The AMPs chosen were ones that killed bacteria by interfering with host metabolism rather than lysing cells to avoid issues caused by the release of endotoxin as Gram-negative *E. coli* are killed. They tested various combinations on *E. coli* in cultured bacteria. Then they took the most effective AMP-containing phagemid and tested the phages it produced using a mouse model of *E. coli* peritonitis. Mice treated with AMP-containing phages had an 80% survival rate compared to the no-phage, control mice that only had a 25% survival rate, while mice treated with unmodified phages had a 50% survival rate.
- Lemon and colleagues [83] added the IDR-1018 AMP gene to the *E. coli* phage T7 genome. IDR-1018 is a 12-amino-acid synthetic derivative of the bovine cathelicidin bactenecin AMP [84]. They found the AMP expressing T7 phages were more effective at killing planktonic and biofilm-contained *E. coli*.
- Ludwig and colleagues [85] added two AMP genes, derived from apidaecin (an AMP originally found in honeybees) to bacteriophage T7. They initially found that expression levels were very low, too low to show any effect on cell killing. In a follow-up study [86], they showed that adding a secretion signal to the apidaecin gene improved expression by preventing the AMP from accumulating inside the infected cells. This phage showed better killing of bacteria as did phages expressing CRAMP and melittin AMPs. These AMP producing phages were not tested for broader host range but were shown to have reduced levels of phage-resistant bacteria, presumably due to killing by the secreted AMP.
- Jo and colleagues [87] modified a newly isolated bacteriophage, pST_PMR_01, which infects *Salmonella enterica* Typhimurium, to express the human antimicrobial peptide LL37 on its capsid. LL37 kills bacteria by disrupting the cell membrane. They found a 10-fold decrease in the rate of bacteriophage-insensitive bacteria produced after infecting *Salmonella* with the modified phage. They suggest that this shows that the phage and the AMP were independently killing bacteria. This was supported by experiments that showed that AMP-containing phage capsids (AMP-phage ghosts) were as protective as the complete AMP-phage in a cultured epithelial cell model. The AMP-phage were also more protective using a *Galleria mellonella* larvae model. As with the phages made by Ludwig and colleagues, the host range of these AMP-phages was not determined.

#### 4.3.4. Other Molecules

The small-acidic spore proteins (SASP) found in *Bacillus subtilis* spores are DNA binding proteins that affect the topology of DNA and are essential for sporulation [88]. When expressed in other species, the proteins block gene expression and DNA replication [89]. Fairhead [90] modified the *Staphylococcus aureus* phage phi11 to include a SASP gene. The SASP gene replaced the phage holin, rendering the modified phage unable to lyse cells which minimized release of the SASP protein and any toxins associated with the target bacteria [91]. The modified phage, SASPject PT1.2, displayed good host range against several hundred *S. aureus* isolates and good specificity, not infecting any strains of nine other *Staphylococcus* species. The modified phage also showed good inactivation kinetics of *S. aureus* in vitro. This phage was being developed for phage therapy by Phico Therapeutics along with SASPject phages, targeting *P. aeruginosa*, *Klebsiella pneumoniae* and *E. coli* [92]; however, the company was liquidated in 2024 [93]. The phages are continuing to be developed by SASPiPhi (https://saspiphi.com/index.html (accessed on 15 July 2026)), which has one phage product targeting *P. aeruginosa* in cystic fibrosis patients about to enter phase 1a/2b clinical trials (https://saspiphi.com/pipeline.html (accessed on 15 July 2026)).

### 4.4. Anti-CRISPR or Other Defensive Proteins

Bacteriophages have evolved proteins to interfere with Cas proteins to protect themselves from this bacterial defense system as well as other defense systems. These bacteriophage proteins can be described as anti-immunity or immunity evasion proteins. Evading these immunity systems would expand the phage’s host range to include bacteria relying on these mechanisms for protection. Table 3 lists some examples of these proteins.

Additional examples of phage anti-immunity proteins can be found in [104,105]. These authors and others have suggested that these immunity evasion proteins could be added to phages to make them more effective phage therapy agents by minimizing the appearance of bacteriophage-resistant bacteria. To date, we are not aware of any studies that have used phages modified in this way in phage therapy trials.

## 5. Phage Steering

Another application of host range changes is to use sensitivity or resistance to a phage, that is, being in or outside of the phage’s host range, to drive another phenotype of the bacterium such as antibiotic resistance or sensitivity. This can occur when the surface molecule used as a receptor for the phage is also the molecule responsible for the antibiotic resistance. Phage steering grew out of observations that found a correlation between changes in phage sensitivity and antibiotic sensitivity. Davies and Reeves [106], for example, noted that *E. coli* resistant to colicins could be divided into two groups sensitive to different colicins and that each group showed a different sensitivity profile to a group of eight phages. German and Misra [107] later showed that spontaneous phage-resistant mutants also had altered sensitivity to a colicin, confirming that the phage receptor and the resistance molecule, in this case the TolC efflux pump, were the same molecule. Burmeister and colleagues [108], using a different phage, also found that *E. coli* with TolC mutations that made the bacteria resistant to the phage also showed decreased antibiotic resistance. This was not true of all the TolC mutations. A few were more antibiotic-resistant, highlighting the diversity of possible mutations of this gene.

In the context of phage therapy, the goal of this application is to use phages to replace a population of phage-sensitive, antibiotic-resistant bacteria with a phage-resistant, antibiotic-sensitive population.

- Chan and colleagues [109] isolated novel phages infecting *P. aeruginosa* and screened for ones that used OprM, a cell surface component of a multi-drug resistance efflux pump system as their receptor. They found one of the isolated phages used OprM as a receptor. They then isolated phage-resistant mutant bacteria and demonstrated that these bacteria were more sensitive to four different antibiotics. Moreover, they found a range in phage sensitivity and antibiotic sensitivity among the mutant bacteria. Bacterial strains with less phage sensitivity (as measured by efficiency of plating (EOP)) also had less antibiotic resistance. In a later paper (Chan et al. [110]) they report using this same phage to treat a patient with a prosthetic vascular graft *P. aeruginosa* infection in the heart. The patient was treated with the phage and then with an antibiotic which resolved the infection.
- Koderi Valappil and colleagues [111] also studied the potential of phages to drive multi-drug-resistant *P. aeruginosa* to drug sensitivity. They isolated novel *P. aeruginosa* phages and identified one that used the MexXY-OprM efflux pump as a receptor. They used this phage in a co-evolution experiment, isolating mutant bacteria that were resistant to the phage. With extended incubation, phage-resistant bacteria with point mutations or deletions in the *mexY* gene were found. These bacteria were also more sensitive to several antibiotics. They also showed that a combination of phage and antibiotic used to treat mice in a lung infection model had a better survival rate than either phage or antibiotic alone.
- Gao and colleagues [112] used a cocktail of four phages to study loss-of-fitness in mutants of *Salmonella enterica* serovar *Enteritidis* that were resistant to the cocktail or to single phage components. One of the phages in the cocktail used a component of the AcrAB-TolC efflux pump as its receptor and some of the phage-resistant bacterial mutants were more sensitive to multiple antibiotics. They also showed that the four-phage cocktail was more effective at treating mice infected with *S. Enteritidis* than each of the phages alone.

## 6. Conclusions

With increasing levels of antibiotic-resistant bacterial pathogens being found world-wide, phage therapy is increasingly seen as an alternative or augmentation to antibiotic treatments of bacterial pathogens. Host range is an essential property of phages that must be determined to see if a phage is suitable for phage therapy. For personalized phage therapy, a narrow-host-range phage can be suitable as long as the patient’s pathogen has been isolated and is infectible by the phage. For phage cocktails that are intended to treat many patients who may be infected by varying strains of a pathogen, phages with broader host ranges are desirable. While there is a vast diversity of phages in the world, phages appropriate to the bacteria infecting a specific patient or group of patients may not be available. Instead of searching for new phages, adapting already-isolated phages to convert them to phages that can infect a previously phage-resistant bacterium is becoming a more viable option using the methods described here. What method is used will depend on the type of phage therapy being done. For example, phage adaptation or the Appelmans protocol would be more useful to obtain a phage to infect a specific pathogen strain. In contrast, a genetically modified phage whose receptor binding protein has been modified to bind to a widespread surface molecule found on many strains might be more suitable for developing a phage cocktail for broad use. These practices are not theoretical. Some of these techniques have already been employed clinically to develop phages that can be used to treat individual patients [28] and to improve phage cocktails [77,79]. An open question is to what degree a phage can be altered and still be considered the same phage in terms of regulation. The Pyophage cocktail made by the Eliava Institute has been refreshed over time by adding phages, including some modified using the Appelmans protocol, but is still considered the same product [31,113]. This may not be true under other countries’ regulatory systems, though. It is clear, however, that host range alteration techniques will continue to play a role in phage therapy as it continues to be developed, tested, and used to treat patients.

## Figures and Tables

**Table 1:** Overview of methods used to alter bacteriophage host ranges.

Method	Genetic Mechanisms	Description	Advantages	Disadvantages
Phage adaptation	Random mutation	Searching for rare plaques that show growth on resistant hosts	Simple	Appearance of mutants is random
Appelmans protocol	Random mutation	Sequential exposure of a phage cocktail to resistant hosts	Directed to specific resistant hosts	Requires phages to have at least limited growth on resistant hosts
Genetic engineering—receptor binding proteins	Genetic engineering and targeted mutagenesis	Controlled exchange or modification of receptor binding proteins	More specific changes can be made Hybrid phages can be made	Not all phages have genetic engineering systems or identified receptor binding proteins
Genetic engineering—gene addition	Genetic engineering and gene insertion	Adding genes that enhance the killing ability of a phage	Enhanced killing can include bacteria outside of phage’s host range Temperate phage can be used if genome entry is sufficient to kill cell	Not all phages have genetic engineering systems. Inserted gene may not be compatible with phage reproduction
Phage steering	Random mutation	Using phage to limit the development of antibiotic resistance in bacteria by selective killing of resistant cells	Directly affects antibiotic-resistant bacteria	Limited to phages that use receptors that are also responsible for resistance to antibiotics

**Table 2:** Representative genetic engineering approaches to modify phages to affect host range.

Method	Description	Advantages	Limitations	Examples
Cloning genes into plasmids with recombination to reinsert into phages	Genes are cloned using PCR or other methods into plasmids. The gene may be modified using site-specific mutagenesis. Cells with the plasmid are infected with the phage and the modified gene is transferred to the phage genome by recombination	Well understood and reliable mechanisms Allows for precise sequence changes	Bacteria must be amenable to transformation to put plasmids into cell Recombination efficiency for small cloned genes can be inefficient requiring screening large numbers of phages in the absence of selection	[41,42,43]
Phagemid or episome modification	During lysogeny, bacterial genome is a dsDNA plasmid and amenable to modification as other plasmids	Same as cloned genes in plasmids	Limited to filamentous phage and temperate phage that do not integrate their genome into the host genome	[44,45]
Recombineering and BRED (Bacteriophage Recombineering of Electroporated DNA)	A dsDNA or ssDNA fragment is introduced into cells and native or cloned recombination enzymes incorporate the guide fragment into the target sequence in infecting phage or prophages	Can be used to insert new genes or mutagenize genes Target gene cloning is not needed as guide fragments can act on infecting phage genomes Can be combined with CRISPR-Cas for improved targeting (CRISPY-BRED)	Recombination frequency can be low so selection screening or a counter-selection against the wild-type sequence is needed Recombination enzymes not found in all species. Added genes may not be compatible with host	Recombineering: [46] BRED: [47] CRISPY-BRED: [48]
CRISPR-Cas guided gene modification	Synthetic CRISPR gene guides Cas protein to cut phage genome at a particular site in the target gene. This can lead to a gene knockout or, if a cloned gene segment is present in the cell, the segment can guide the insertion or mutation of sequences	Can modify any unique sequence Cas gene can be coded from a gene on a plasmid so method can be used with any host that can be transformed	Depending on the gene and system, it may be necessary to screen phages for ones with the desired mutation Host must be transformable	[49,50]
Phage genome assembly in vitro and rebooting in bacteria	Phage genome can be assembled from fragments and transfected into bacteria to produce virion particles (rebooting)	Genome fragments made by PCR or by synthetic nucleic acid synthesis can contain multiple modifications of phage genes Scarless assembly methods such as Gibson or Golden Gate assembly can be used to generate genomes from fragments	Transfection efficiency may be low	[51,52] Ref. [53] describes a similar method in which phage genomes are assembled in yeast as yeast artificial chromosomes, isolated, and transfected into bacteria for rebooting
Cell-free transcription-translation systems (TXTL systems)	Assembled phage genome is mixed with TXTL mix. After incubation, product virions are used to infect host cells to grow phages	TXTL systems are commercially available Genome assembly from fragments uses same methods as above	Virion assembly cannot require intact host cells or host enzymes	[54]

**Table 3:** Examples of phage proteins that protect phages from various host defense systems.

Protein(s)	Source	Defense System Targeted	Reference
Dar proteins	*E. coli* phage P1	Restriction	[94]
Methyltransferases	Multiple phages	Restriction	[95]
S-adenosyl-l-methionine lysase (SAMase)	*E. coli* phage T3	Restriction	[96]
JSS1_004 protein kinase	*Salmonella* phage JSS1	DnD system (DNA degradation system)	[97]
Acr proteins	Multiple phages	CRISPR-Cas	[98,99]
Anti-Tmn protein	*E. coli* phage φSMS22	Tmn (transmembrane NTPase) system	[100]
Dmd protein	*E. coli* phageT4	Toxin-antitoxin	[101]
Acb1	*E. coli* phage T4	CBASS-induced abortive infection	[102]
Apyc	*B. subtilis* phage SBSphiJ	Pycsar-induced abortive infection	[102]
Aqs1	*Pseudomonas* phage DMS3	Quorum sensing control of multiple defense systems	[103]

## Data Availability

No new data were created or analyzed in this study. Data sharing is not applicable to this article.
